# Plasma EBV DNA: A Promising Diagnostic Marker for Endemic Burkitt Lymphoma

**DOI:** 10.3389/fonc.2021.804083

**Published:** 2021-12-14

**Authors:** Rena R. Xian, Tobias Kinyera, Isaac Otim, Joshua N. Sampson, Hadijah Nabalende, Ismail D. Legason, Jennifer Stone, Martin D. Ogwang, Steven J. Reynolds, Patrick Kerchan, Kishor Bhatia, James J. Goedert, Sam M. Mbulaiteye, Richard F. Ambinder

**Affiliations:** ^1^ Department of Pathology, Johns Hopkins School of Medicine, Baltimore, MD, United States; ^2^ Department of Oncology, Johns Hopkins School of Medicine, Baltimore, MD, United States; ^3^ EMBLEM Study, African Field Epidemiology Network, Kampala, Uganda; ^4^ Department of Pediatrics, St. Mary’s Hospital Lacor, Gulu, Uganda; ^5^ Division of Cancer Epidemiology and Genetics, National Cancer Institute, National Institutes of Health, Bethesda, MD, United States; ^6^ Division of Intramural Research, National Institute of Allergy and Infectious Diseases, National Institutes of Health, Bethesda, MD, United States; ^7^ Children’s Ward, Kuluva Hospital, Arua, Uganda

**Keywords:** Epstein-Barr virus, Burkitt lymphoma, malaria, plasma DNA, cell-free DNA

## Abstract

Endemic Burkitt lymphoma (eBL) is the most common childhood cancer in regions of equatorial Africa where *P. falciparum* malaria is holoendemic. The tumor is consistently associated with Epstein-Barr virus (EBV). Screening for EBV DNA in plasma in a high-risk population in Hong Kong has been shown to be useful in facilitating the early diagnosis of nasopharyngeal carcinoma, another EBV-associated tumor. Here, we investigate plasma EBV as a diagnostic marker for eBL in children in Uganda. We studied plasma specimens from 25 children with eBL and 25 controls matched for age (<3-16 years), gender and geography, including many with asymptomatic *P. falciparum* infection. These specimens were previously collected under the auspices of the EMBLEM (Epidemiology of Burkitt lymphoma in East African children and minors) study. After cell-free DNA isolation, plasma EBV DNA was measured using a quantitative PCR assay that amplifies the large internal repeats of the EBV genome. All children with eBL had measurable plasma EBV, as compared to 84% of control children. The median plasma EBV DNA level was 5.23 log_10_ copies/mL (interquartile range 3.54-6.08 log_10_ copies/mL) in children with eBL. In contrast, the median plasma EBV DNA level was 0.37 log_10_ copies/mL (interquartile range 0.18-1.05 log_10_ copies/mL) in children without lymphoma. An EBV threshold of 2.52 log_10_ copies/mL yielded a sensitivity of.88 and a specificity of 1. The estimated AUC was 0.936 (95% CI: 0.8496 – 1.00) for the corresponding ROC curve. Plasma EBV copy number did not depend on age, gender, or malaria screening status. However, two control children with asymptomatic *P. falciparum* infection and parasitemia also had high plasma EBV copy number. Our analysis suggests that measurements of EBV copy number in plasma may be useful in identifying children with eBL versus control children. A promising area for future research is the differentiation of high copy number associated with tumor versus high copy number associated with asymptomatic parasitemia.

## Introduction

Epstein-Barr virus (EBV) was first discovered in cell lines established from Burkitt lymphoma (BL) tumors from Uganda (1, 2). At first, it appeared that the distribution of the tumor might correspond to the distribution of the virus, but ultimately it was appreciated that the tumor occurs relatively commonly (~5/100,000) in children in certain regions of equatorial Africa where it is described as endemic Burkitt Lymphoma (eBL), and sporadically elsewhere (~0.1/100,000) (3). In contrast, the virus is ubiquitous in all populations irrespective of geography. In areas with eBL, the lymphoma is almost always associated with EBV, but this association decreases to less than 30% (4) in sporadic BL (sBL). It should also be noted that eBL in Africa corresponds fairly closely with the distribution of holoendemic *P. falciparum* malaria (5–7).

As an oncogenic virus, EBV is also associated with undifferentiated nasopharyngeal carcinoma (NPC). As with BL, there are pronounced geographic differences in the incidences of NPC (~3/100,000 in Southern China vs. ~0.4/100,000 in the United States) (8) leading to a similar paradigm of endemic vs. sporadic disease. Unlike BL, the EBV association is very strong in both high and low incidence regions. Given this strong association, plasma EBV DNA has been extensively studied as a diagnostic marker for NPC. A large study in Hong Kong screened approximately 20,000 men at high risk for NPC by PCR for EBV DNA in plasma (9), and found more than 300 cases of NPC, most of which represented early-stage disease that could be treated with radiation to achieve long term survival.

The diagnostic utility of blood-based EBV DNA measurements has also been explored in eBL. Several previous investigators have reported high EBV DNA copy numbers in whole blood, buffy coat, peripheral blood mononuclear cells, and plasma from children with eBL (10–12). With recent increased appreciation of circulating cell-free tumor DNA as a tumor marker for many cancers, including lymphoma (13–15), and the success of the Hong Kong approach for NPC, we studied EBV copy number in plasma in children from Uganda with and without eBL.

## Methods

### Patient and Specimen Selection

Under the auspices of the EMBLEM (Epidemiology of Burkitt lymphoma in East African children and minors) study, blood specimens were collected from children with presumptive eBL and from population controls that were frequency-matched on age, sex, and geographical region as described (3, 16). The EMBLEM study was conducted with ethical approval from the Uganda Virus Research Institute Research and Ethics Committee, the Uganda National Council of Science and Technology (HS-816) and the National Cancer Institute Special Studies Institutional Review Board (10-C-N133). Written informed consent was obtained from the parents or guardians of the children and written informed assent was obtained from children aged seven years or older prior to enrolment.

Cases of eBL were predominantly defined histologically or cytologically. When this was not possible, eBL was defined according to clinical features, imaging and laboratory results that supported the presumptive diagnosis of eBL. The ages of the children were 1-16 years. Venous blood specimens were obtained in EDTA tubes. Blood specimens were examined by light microscopy (thick film) for asexual parasite forms and for *plasmodium* antigen using a commercial rapid diagnostic test (17). Research blood specimens were transported in cold boxes to local laboratories where plasma was separated by centrifugation and stored at−80°C.

For the present investigation only specimens from children from Uganda were studied. Case selection focused on sampling a variety of age groups with an equal admixture of male and female patients, and screen status for asymptomatic antigenemia and/or parasitemia. Controls were then selected to match the distribution of the cases. Frozen plasma was obtained and cell-free DNA was isolated from 500 µL of plasma using the QIAamp DNA blood mini kit (Qiagen Inc, Valencia, CA, USA) according to manufacturer instructions.

### EBV Measurements

After DNA isolation, quantitative EBV PCR was performed using a primer and probe set corresponding to the BamH-W region of the EBV genome (5’-CCCAACACTCCACCACACC-3’, 5’- TCTTAGGAGCTGTCCGAGGG-3’, 5’-(6-FAM) CACACACTACACACACCCACCCGTCTC (BHQ-1)-3’) as previously described (18). Namalwa DNA (Namalwa cell line genomic DNA, ATCC #CRL-1432) was used for calibration and quantification of EBB copy number. EBV copy number was measured as absolute copies per mL of plasma (copies/mL).

### Statistical Methods

We log_10_ transformed the EBV copy number and illustrated the difference in log-transformed levels between cases and controls by a box plot and formally evaluated the difference using a Wilcoxon rank sum test. This analysis was then repeated in subgroups defined by demographic and clinical characteristics. To further evaluate the ability of plasma EBV DNA copy number to distinguish eBL from controls, we performed a Receiver Operator Characteristics (ROC) analysis and its 95% confidence interval (CI), and calculated the Area Under the Curve (AUC) and associated 95% CI. The CI for the ROC and AUC were calculated by the bootstrap procedure. Finally, we evaluated if demographic and clinical characteristics were associated with log-transformed levels in cases vs. controls using a Wilcoxon rank sum test. All statistical analyses were performed using the R programming language (v4.1.1) (19) and JMP (v16.0.0, SAS Institute Inc., Cary, NC). Statistical significance for all tests was defined as p-value < 0.05.

## Results

We studied 25 children with eBL and 25 children without lymphoma ranging in age from less than three years of age up to 16 years of age. There was an approximate equal proportion of males and females, as well as an approximate equal proportion of children screening positive for *p. falciparum*. None of the subjects had symptomatic malaria infection at the time of enrollment. Characteristics of the study populations are listed in **Table 1**. EBV *in situ* hybridization was only performed in 3 tumors, of which 2 were EBV positive.

**Table 1:** Demographics and clinical characteristics.

	eBL (%)	Control (%)
Total	25 (100%)	25 (100%)
Gender		
Male	13 (52%)	13 (52%)
Female	12 (48%)	12 (48%)
Age range (years)		
<3	1 (4%)	1 (4%)
3-<6	7 (28%)	7 (28%)
6-<9	5 (20%)	5 (20%)
9-<12	7 (28%)	7 (28%)
12-<16	5 (20%)	5 (20%)
Malaria antigen screen		
Negative	14 (56%)	11 (44%)
Positive	11 (44%)	14 (56%)
Parasitemia		
No evidence	14 (56%)	11 (44%)
Recent evidence	0 (0%)	3 (12%)
Current evidence	11 (44%)	11 (44%)

**Figure 1:**
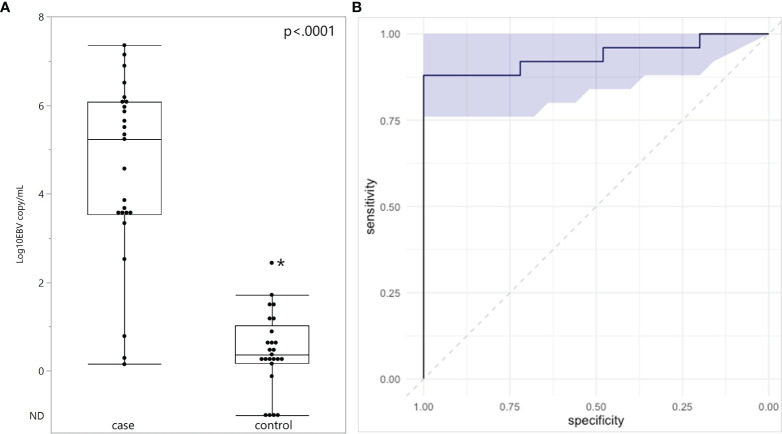
Plasma EBV quantification in endemic Burkitt Lymphoma. **(A)** Plasma EBV levels in 25 children with eBL and 25 controls. ND, Not Detected. **(B)** ROC analysis curve based on EBV quantification and shaded area represents 95% confidence intervals. *Indicates outlier.

Plasma EBV DNA was quantified in all subjects (**Figure 1A**). EBV DNA was detected in every plasma specimen from eBL children. The median plasma EBV DNA level was 5.23 log_10_ copies/mL with an interquartile range of 3.54-6.08 log_10_ copies/mL. Of the three children with eBL, for whom EBV *in situ* hybridization data were available, the two children with EBV-positive eBL had EBV copy numbers of 5.86 log_10_ and 3.63 log_10_ copies/mL. The one child with an EBV-negative tumor only had 0.78 log_10_ copies/mL in plasma. In children without eBL, EBV DNA was detected in 21 of the 25 control children (84%). The median plasma EBV DNA level was 0.37 log_10_ copies/mL with an interquartile range of 0.18-1.05 log_10_ copies/mL. EBV copy number was higher in eBL cases, as compared to controls (p<.0001). Next, we evaluated the diagnostic accuracy of plasma EBV levels by performing a ROC analysis (**Figure 1B**). There was high discriminatory accuracy with an AUC of 0.936 (95% CI: 0.8496 – 1.00) for the corresponding ROC curve. Of note, using a threshold of 2.52 log_10_ copies/mL, the sensitivity of plasma EBV copy number for eBL was.88 and the specificity was 1. Outlier analysis of children without eBL showed that high EBV copy number (2.44 log_10_ copies/mL) was found in a single child, who also had demonstrable parasites in blood.

**Figure 2:**
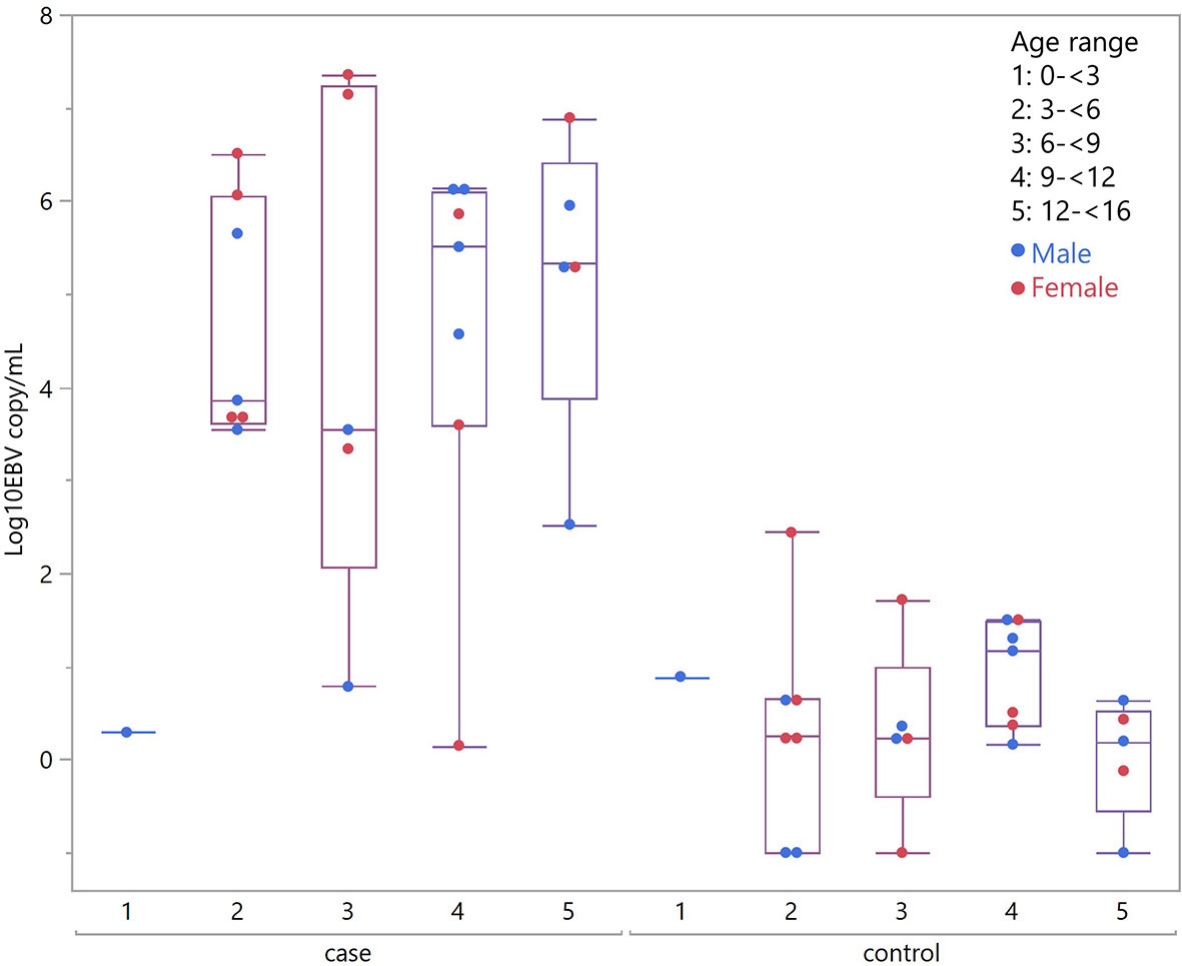
Plasma EBV quantification by age group. Plasma EBV levels in eBL (p = .3559) and in controls (p = .6488) based on age range and gender.

**Figure 3:**
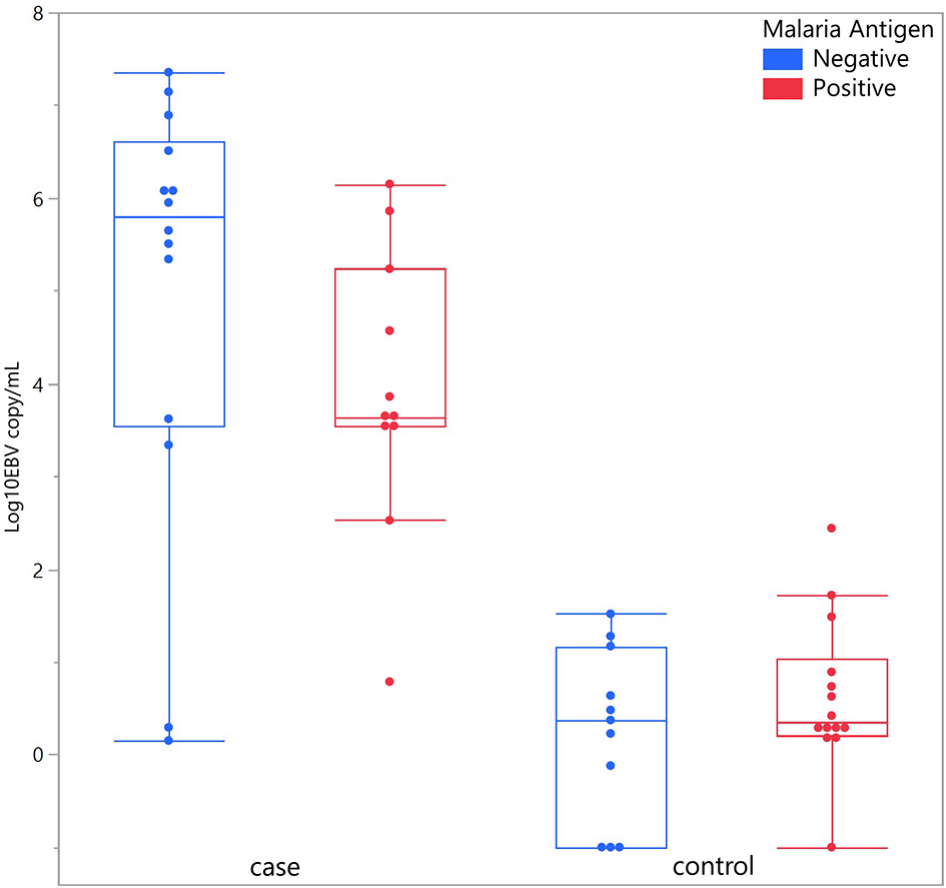
Plasma EBV quantification based on malaria antigen screen status. Plasma EBV levels in eBL (p = .0950) and controls (p = .5463) based on malaria antigen screening status.

Considering children with eBL and those without, there were no differences in plasma EBV detection rate based on age, gender or malaria screening status (either antigenemia or parasitemia). In particular, among children without eBL, there were no differences in EBV detection rate based on parasitemia status. Ninety-one percent (10/11) of children with asymptomatic *P. falciparum* infection, as demonstrated by visualized circulating parasites, tested positive for plasma EBV, and 79% (11/14) of children without parasitemia also tested positive for plasma EBV. As for plasma EBV levels, there were also no differences in plasma EBV copy number within cases or controls based on age or gender (**Figure 2**), or malaria screening status (either antigenemia or parasitemia) (**Figure 3**) suggesting plasma EBV levels was not modified by either of these factors for children with eBL, nor children without lymphoma. Among children with eBL, EBV copy number was slightly lower in children with asymptomatic current parasitemia vs. those without current parasitemia (median 3.63 log_10_ copies/mL vs. median 5.82 log_10_ copies/mL, p=.0950). Among children without eBL, there was also no difference in EBV copy number (median 0.37 log_10_ copies/mL vs. median 0.35 log_10_ copies/mL, p=.5463) based on malaria status. Confirmation that plasma EBV copy number was not related to gender or malaria screening status was confirmed by subgroup analysis (**Table 2**) where children with the same modifying characteristics and eBL were compared to those without lymphoma. This analysis showed that irrespective of gender or malaria status, plasma EBV DNA levels remained a robust marker that can distinguish cases from controls.

**Table 2:** Plasma EBV levels based on clinical characteristics.

		eBL	Control	p-value
		Log_10_ EBV copy/mL	Log_10_ EBV copy/mL	
All	median	5.23	0.37	<.0001
	mean	6.34	1.27	
	min	0.15	0.00	
	max	7.35	2.44	
Gender				
Male	median	4.57	0.28	.0001
	mean	5.55	0.82	
	min	0.29	0.00	
	max	6.15	1.52	
Female	median	5.65	0.39	.0004
	mean	6.62	1.49	
	min	0.15	0.00	
	max	7.35	2.44	
Malaria antigen screen				
Negative	median	5.82	0.37	.0005
	mean	6.57	0.83	
	min	0.15	0.00	
	max	7.35	1.52	
Positive	median	3.63	0.35	<.0001
	mean	5.33	1.44	
	min	0.78	0.00	
	max	6.15	2.44	
Parasitemia				
No evidence	median	5.82	0.37	.0005
	mean	6.57	0.83	
	min	0.15	0.00	
	max	7.35	1.52	
Recent evidence	median	NA	0.65	NA
	mean	NA	0.74	
	min	NA	0.62	
	max	NA	0.89	
Current evidence	median	3.63	0.25	.0002
	mean	5.33	1.53	
	min	0.78	0.00	
	max	6.15	2.44	

NA, Not Applicable.

## Discussion

Our results show that although EBV DNA is readily detected in children without eBL, the presence of EBV in cell-free plasma DNA at a copy number in excess of 2.52 log_10_ copies/mL (or 334 copies/mL) serves as a reliable indicator of the presence of eBL in children in Uganda. The report from Hong Kong using plasma EBV DNA screening for nasopharyngeal carcinoma suggested that screening of high risk populations facilitated earlier diagnosis and resulted in increased survival rates. In this regard, we note that the cure rate for pediatric BL in high resource settings with chemotherapy is >90% (20, 21). However, in limited resource settings, the cure rate for endemic BL is much lower—44% in a recent report from Uganda (22). In multivariate analysis, the only measure that predicted mortality in Uganda was performance status (e.g. time out of bed, the ability to perform self-care), which almost certainly is a surrogate for CNS involvement and far advanced disease (23). Since standard chemotherapies do not clear central nervous system disease, advanced disease may contribute to the disparity in survival in Ugandan children with eBL. In that report, 16% of patients were unable to perform self-care or were confined to the bed, and 7% died before treatment could be initiated. Earlier diagnosis, before performance status is impaired, would almost certainly improve survival and cure of eBL. Hence, further investigations of the diagnostic utility of EBV DNA detection to facilitate earlier diagnosis in eBL hold great promise in high-risk populations, or children presenting with symptoms suggestive of eBL.

Aspects of the investigations here are similar to 3 previous investigations focused on plasma from East African children with eBL (**Table 3**) (10–12). One of these studies focused on children in Uganda with eBL, malaria, or other minor infection (10). EBV DNA was detected in plasma from children with eBL in 89%, in control children without acute malaria in 18%, and in control children with acute malaria defined as *P. falciparum* parasitemia (≥2500 asexual parasites/µL) in 31% (**Table 3**). These authors assessed the impact of 2 weeks of malaria treatment (sulfadoxine, pyrimethamine and amodiaquine) and found that EBV detection fell to 5% with an 85% clearance rate. In a recent report from Malawi, plasma from 88 children with eBL and 16 children initially suspected to have lymphoma, but pathologically confirmed to have non-lymphoproliferative disorders, were studied (12). EBV DNA was detected in plasma specimens from 86% of eBL children and 12% of the control children. A third study was focused on the relationship between sickle cell trait and eBL and found no association (11). However, they did note that plasma EBV copies in excess of 120,000 copies/mL (or 5.08 log_10_ copies/mL) would provide 90% sensitivity and specificity for eBL when compared to malaria holoendemic controls. Their data showed an AUC of 0.962 for the corresponding ROC curve. Overall, the results of these studies are similar to ours, though our assay is much more sensitive, and show that EBV DNA is consistently detectable in plasma in the great majority of children with eBL, but it is also commonly detected in geographically matched children without eBL.

**Table 3:** Comparison of plasma EBV detection rate in African children.

	Current Report	Donati et al.	Westmoreland et al.
EBV target amplified	Large Internal Repeat	LMP1	EBNA3C
Copies of EBV target	4-12	1	1
eBL % (n)	100% (25/25)	89% (23/26)	86% (76/88)
Malaria (+) control % (n)	73% (8/11)	18% (7/40)	NA
Malaria (-) control % (n)	93% (13/14)	31% (13/42)	NA
Malaria (unknown) control % (n)	NA	NA	12% (2/16)

NA, Not Applicable.

There are differences among these studies that are worth noting. The investigators in each of the prior studies used different sets of primers to amplify EBV DNA (**Table 3**). We used a primer/probe set that targets the large internal repeats, which should yield increased sensitivity since there are 4-12 copies of these repeats in each EBV genome. This strategy and the same target has been used by many other studies to increase sensitivity, including the large screening study from Hong Kong (9, 18, 24). The three other investigations in eBL cited above all targeted single copy genes. While our approach benefited from increased sensitivity, as a function of target copy number, this may come at a cost to precision with regard to estimating copy number. Consistent with this difference, two of the eBL reports indicated a lower detection rate of plasma EBV DNA in eBL than the present report (10, 12), and the third report did not provide the positivity rate in eBL (11).

The percentage detection of plasma EBV may also be influenced by the percentage of tumors that are in fact EBV associated. The gold standard for defining EBV association in tumors is EBER *in situ* hybridization (25, 26). In none of the reports above was EBV-association confirmed by *in situ* hybridization in the majority of specimens. This limitation also applies to the present study. Thus, there remains the possibility that a fraction of the tumors are not in fact EBV-associated, which would correspond to a very low or undetectable EBV copy number, as in the single case in our report.

With regard to EBV DNA in plasma from control children, the previous studies differ from the present with regard to the characteristics of these children without lymphoma. We enrolled population-based controls of a similar age and geographic distribution as the eBL cases studied who were asymptomatic for malaria. In the other studies, the malaria-negative control group consisted of children who visited a general clinic with mild symptoms unrelated to malaria who did not have parasites detected in blood smears (10), or consisted of children suspected of having lymphoma, but without lymphoproliferative disease on biopsy (12). It is not clear how these differences in selection of controls might be expected to impact on the frequency of EBV detection in plasma in control groups.

With respect to controls with high EBV copy number, we found two such children who had asymptomatic current parasitemia and copy numbers (2.43 log_10_ and 1.72 log_10_) approaching those in eBL. Others have also reported this phenomenon (10, 11). Both of these investigations used digestion with DNaseI to distinguish virion EBV DNA, which might be protected from digestion by the viral envelope, from cell-free EBV DNA released from infected cells. Both publications noted that the interpretation of the assay results were difficult insofar as the process of freezing and thawing may render virion DNA susceptible to DNaseI digestion. Nonetheless both publications suggested there may be DNase I-resistant EBV viral genomes in controls. A subset of this viral DNA may reflect lysis of latently infected blood cells *in vivo* or *ex vivo* (i.e. after collection in blood collection tubes). It is now well established that collection of plasma from EDTA tubes is associated with substantial cell lysis *ex vivo* and that the resulting contamination of circulating cell-free DNA with cellular DNA often obscures the characteristics of the cell-free DNA analyses (27). Cell stabilizing blood collection tubes have since been developed that largely overcome this issue, but were not employed for collecting blood in any of these investigations (28). If most of the viral DNA detected in children with malaria is the result of *ex vivo* lysis, then the use of cell stabilizing blood collection tubes might improve differentiation of EBV tumor DNA from EBV DNA in controls with or without malaria. Alternatively, if most of the EBV DNA detected in plasma from children with malaria is in fact virion DNA, assessment of CpG DNA methylation might differentiate virion DNA, which is never methylated, from other cellular DNA (29).

Our analysis suggests that measurements of EBV copy number in plasma may be a promising diagnostic marker for endemic Burkitt lymphoma in Africa and BL occurring in other regions, such as Latin America (30), with high proportions of EBV(+) BL. These results warrant exploration in larger studies, which would further characterize the clinical scenarios that might benefit most for such plasma DNA assessments. Additional areas of future investigations could also focus on the source of high level plasma EBV DNA in children without eBL, and differentiating high copy number associated with tumor versus high copy number associated with asymptomatic parasitemia furthering refining the accuracy of EBV measurements for eBL.

## Data Availability Statement

The raw data supporting the conclusions of this article will be made available by the authors, without undue reservation.

## Ethics Statement

The EMBLEM study was conducted with ethical approval from the Uganda Virus Research Institute Research and Ethics Committee, the Uganda National Council of Science and Technology (HS-816) and the National Cancer Institute Special Studies Institutional Review Board (10-C-N133). Written informed consent was obtained from the parents or guardians of the children and written informed assent was obtained from children aged seven years or older prior to enrolment. Written informed consent to participate in this study was provided by the participants’ legal guardian/next of kin.

## Author Contributions

RX, SM, KB, and RA contributed to conception and design of the study. JNS performed the EBV measurements and performed data analysis along with RX and RA. RX and JNS performed the statistical analyses. SM, MO, SR, and PK supervised the fieldwork. RX, RA, and SM wrote the first draft of the manuscript. IL, IO, PK, HN, MO, KB, SR, and JG conducted and monitored fieldwork. All authors contributed to manuscript revision, read, and approved the submitted version.

## Funding

This work was supported by R21CA232891, R01CA250069, and P30CA06973. The EMBLEM study was funded by the National Cancer Institute, National Institutes of Health, under Contract No. HHSN261200800001E, Contract No. HHSN261201100063C, and Contract No. HHSN261201100007I (Division of Cancer Epidemiology and Genetics), and in part (SJR) by the Division of Intramural Research, National Institute of Allergy and Infectious Diseases, National Institutes of Health.

## Author Disclaimer

The content of this publication does not necessarily reflect the views or policies of the Department of Health and Human Services, nor does mention of trade names, commercial products or organizations imply endorsement by the U.S. Government.

## Conflict of Interest

The authors declare that the research was conducted in the absence of any commercial or financial relationships that could be construed as a potential conflict of interest.

## Publisher’s Note

All claims expressed in this article are solely those of the authors and do not necessarily represent those of their affiliated organizations, or those of the publisher, the editors and the reviewers. Any product that may be evaluated in this article, or claim that may be made by its manufacturer, is not guaranteed or endorsed by the publisher.
